# Standardised arthropod (Arthropoda) inventory across natural and anthropogenic impacted habitats in the Azores archipelago

**DOI:** 10.3897/BDJ.9.e62157

**Published:** 2021-03-10

**Authors:** José Marcelino, Paulo A. V. Borges, Isabel Borges, Enésima Pereira, Vasco Santos, António Onofre Soares

**Affiliations:** 1 cE3c – Centre for Ecology, Evolution and Environmental Changes / Azorean Biodiversity Group and Universidade dos Açores, Rua Madre de Deus, 9500, Ponta Delgada, Portugal cE3c – Centre for Ecology, Evolution and Environmental Changes / Azorean Biodiversity Group and Universidade dos Açores, Rua Madre de Deus, 9500 Ponta Delgada Portugal; 2 cE3c – Centre for Ecology, Evolution and Environmental Changes / Azorean Biodiversity Group and Universidade dos Açores, Rua Capitão João d’Ávila, São Pedro, 9700-042, Angra do Heroismo, Portugal cE3c – Centre for Ecology, Evolution and Environmental Changes / Azorean Biodiversity Group and Universidade dos Açores, Rua Capitão João d’Ávila, São Pedro, 9700-042 Angra do Heroismo Portugal; 3 IUCN SSC Mid-Atlantic Islands Specialist Group, Angra do Heroísmo, Portugal IUCN SSC Mid-Atlantic Islands Specialist Group Angra do Heroísmo Portugal

**Keywords:** Arthropoda, Azores, São Miguel, Terceira, Flores, Santa Maria, Pico, Island, anthropogenic impact gradient, habitat types

## Abstract

**Background:**

In this paper, we present an extensive checklist of selected arthropods and their distribution in five Islands of the Azores (Santa Maria. São Miguel, Terceira, Flores and Pico). Habitat surveys included five herbaceous and four arboreal habitat types, scaling up from native to anthropogenic managed habitats. We aimed to contribute to the ongoing effort to document the terrestrial biodiversity of the world, in particular the Portuguese archipelago of the Azores, as islands harbour a significant portion of unique terrestrial biodiversity. Selection of Arthropoda groups for the current checklist was based on their known richness and abundance (Arachnida, Collembola, Hemiptera, Neuroptera, Coleoptera, Hymenoptera), in almost all terrestrial ecosystems, as well as their importance in current Integrated Pest Management and alternative Biocontrol protocols at large (i.e. hymenopteran parasitoids and beneficial Coleoptera). In addition, we include the list of Dermaptera, Orthoptera, Psocoptera and Thysanoptera species. These assembled groups represent part of the monitoring programme EDEN Azores (2008-2014), where all Arthropod fauna, at all strata, within nine representative habitats of the abovementioned five Islands of the Azores was recorded.

**New information:**

In this study, a total of 116,523 specimens, belonging to 483 species and subspecies of selected groups of arthropods, are reported by order, family and, when possible, genus and species. Hymenopteran, mostly parasitoids, accounted for the most represented taxa across all the monitoring and sampling phase of EDEN Azores (193 species and mophospecies), followed by Coleoptera (95 species); Collembola (89 species); and Araneae (72 species).

A total of 37 non-native species are reported for the first time in the Azores. **Coleoptera**: *Asaphidion flavipes* (Linnaeus, 1761) (Carabidae); *Tachyporus dispar* (Paykull, 1789) (Staphylinidae). **Hemiptera**: *Acrosternum heegeri* Fieber, 1861 (Pentatomidae). **Collembola**: *Entomobrya regularis* Stach, 1963 (Entomobryidae); *Lepidocyrtus lusitanicus piezoensis* (Simón-Benito, 2007) (Entomobryidae); *Jordanathrix articulata* (Ellis, 1974) (Sminthuridae); *Sminthurinus quadrimaculatus* (Ryder, 1879) (Katiannidae); *Himalanura* sp. (Entomobryidae); *Protophorura* sp. (Onychiuridae). **Hymenoptera, parasitoids**: *Aphidius colemani* Viereck, 1912 (Braconidae); *Aphidius ervi* Haliday, 1834 (Braconidae); *Aphidius matricariae* Viereck, 1912 (Braconidae); *Aphidius rhopalosiphi* Stefani-Perez, 1902 (Braconidae); *Aphidius rosae* (Haliday, 1834) (Braconidae); *Aphidius urticae* Haliday, 1834 (Braconidae); *Centistidea ectoedemiae* Rohwer, 1914 (Braconidae); *Meteorus unicolor* (Wesmael, 1835) (Braconidae); *Meteorus collaris* (Spin.) Hal. – Ruschka, Fulmek, 1915 (Braconidae); *Orthostigma cratospilum* (Thomson, 1895) (Braconidae); *Orthostigma latriventris* Ratzeburg, 1844 (Braconidae); two other species of *Orthostigma* sp.; *Pseudopezomachus bituberculatus* (Marshall, 1905) (Braconidae); *Tanycarpa punctata* (van Achterberg, 1976) (Braconidae); *Gonatopus clavipes* (Thunberg, 1827) (Dryinidae). New genera not previously recorded for the Azores include: *Pycnetron* sp. (Chalcidoidea: Pteromalidae); four species of *Aspilota* sp. (Braconidae: Alysiinae); four species of *Chorebus* sp. (Braconidae: Aphidiinae: Alysiinae); *Microgaster* sp. (Braconidae: Microgastrinae); *Homolobus* sp. (Braconidae: Homolobinae); *Lodbrokia* sp. (Braconidae: Alysiinae).

These 37 taxa were found in several Islands and five are new species for Flores Island, 10 species are new for Pico Island, 12 species are new for Terceira Island, 19 species are new for S. Miguel Island and five species are new for S. Maria Island.

Additional species records for the Islands included: Flores (5 Collembola, 9 Araneae; 2 Hemiptera; 8 Coleoptera, 8 Hymenoptera), Pico (4 Collembola; 7 Araneae; 4 Hemiptera; 11 Coleoptera; 9 Hymenoptera), Terceira (4 Collembola; 1 Araneae; 3 Hymenoptera), S. Miguel (1 Araneae; 2 Coleoptera; 3 Hymenoptera), S. Maria (5 Collembola; 3 Araneae; 2 Hemiptera; 2 Hymenoptera).

## Introduction

Biodiversity loss is accelerating at an unprecedented rate (Maxwell et al. 2016, Borges et al. 2019, Wagner et al. 2021), particularly in islands (Whittaker et al. 2017). Current drivers of biodiversity loss include habitat change (i.e. habitat loss, degradation and fragmentation), invasive species, pollution and contamination and climate change (Titeux et al. 2016, Sanchez-Bayo and Wyckhuys 2019). Land-use reconversion is a catalyst for major biodiversity changes in the world, namely in island ecosystems (Russell and Kueffer 2019). The inventory and monitoring of island biodiversity is critical for understanding current and future trends in biodiversity erosion (Borges et al. 2019) as remote archipelagoes enclose high endemism levels and a significant portion of terrestrial biodiversity.

In the current study, we focus in Azores Islands (Portugal) and on its arthropod diversity inventory. Arthropods are recognised as one of the most endangered taxa in the globe, vital for ecosystem stability and food security (Hochkirch 2016, Harvey et al. 2020, Raven and Wagner 2021, Wagner et al. 2021). Composed of nine Islands lying on the North Atlantic Ocean, (39º43'23'' N [Corvo] - 36º55'43'' N [Santa Maria]; 24º46'15'' WG [Formigas islets] - 31º16'24'' WG [Monchique Islet – Flores]), these Islands, when discovered, were completely covered by dense forests (Frutuoso 1978). These forests included *Erica-Morella* woodlands at levelled coastal areas and *Picconia-Morella* forests up to 300 m a.s.l. From 300 m to 600 a.s.l., the sub montane forests dominated (the Azorean Laurel forests, predominatly *Laurus azorica*), which probably covered more than two thirds of the Islands (Elias et al. 2016). Above the *Laurus* forests, between 600 m and 1000 a.s.l., *Juniperus-Ilex* forests and *Juniperus* woodlands would have covered most of the areas (Elias et al. 2016). At higher elevations, *Calluna-Juniperus* scrublands may have covered mountain ridges and *Calluna-Erica* scrublands and *Calluna* scrublands would have occupied Pico Mountain, above 1200 m a.s.l., as they still do today (Elias et al. 2016).

The topography of the Azores is characterised by the presence of numerous catchments, ravines and mainly seasonal water streams. Climate and hydrography, together with remote geographic isolation (i.e. central zone of the North Atlantic) and absence of any close continental masses (the nearest landmasses are Europe > 1300 km away and North America > 3200 km away), as well as the complex marine Current System (Caldeira and Reis 2017), contribute to a temperate climate and high humidity throughout the year. These environmental conditions and a nutrient-rich volcanic soil, still support an abundant flora in spite of intense anthropogenic influence and land reconversion to agriculture and forestry activities. Mixed and pristine forests [predominantly native evergreen Laurel forests (Laurisilva), a humid broadleaf *Laurus azorica* (Seub.) Franco forest] covers many islands’ hillsides (Cardoso et al. 2007, Hortal et al. 2006). Thirteen percent of their land surface is protected (World Heritage, Biosphere and Natura 2000 Networks).

The Azorean Islands have a long history of habitat loss and land-use changes, with only circa 5% of the native forest remaining intact (Borges et al. 2020). Deforestation has occurred extensively, initially at low elevations, but subsequently extended to mid- and higher elevations due to anthropogenic intervention and timber use as an energy source. Currently, six main habitats can be found in Azores, i.e. i) the original native forests, restricted to high elevations with some small pockets at mid-elevations and disturbed mixed vegetation at low elevation; ii) exotic fast-growing tree plantations, dominated by *Cryptomeria japonica*; iii) exotic mixed forests, dominated by the invasive tree *Pittosporum undulatum*; iv) several types of grasslands, including high elevation natural grasslands, although mostly dominated by intensive pastureland at low and mid-elevations and semi-natural pastures at mid- and high elevations; v) native bogs and fens at high elevations; and vi) several types of agro-ecosystems including vineyards, orchards and corn fields.

The Azorean arthropod fauna is well known and includes approximately 2332 species and subspecies, with less than 300 of these being endemic (Borges et al. 2010). Land-use changes had an impact on the composition of Azorean arthropod fauna, now dominated by exotic species, particularly in anthropogenic habitats (Borges et al. 2008), but also, to some extent, in native forest, such as in the case of soil arthropods, particularly Collembola (Cicconardi et al. 2017). Endemic arthropods are mostly restricted to native habitats (Borges et al. 2008, Rigal et al. 2018). However, endemic and native insect pollinators successfully adapted to new anthropogenic habitats and are providing essential ecosystem services in agro-ecosystems (Picanço et al. 2017). The impact of anthropogenic disturbance on vascular plants was also investigated in parallel with the arthropod distribution (Marcelino et al. 2013, Silva et al. 2017), observing that endemic and native plant species are not restricted only to natural habitats, but also occur in human-managed arborescent habitats. Invasive species dominate human-managed habitats, whilst also found at the edges of natural habitats.

## General description

### Purpose

**Figure 1.**
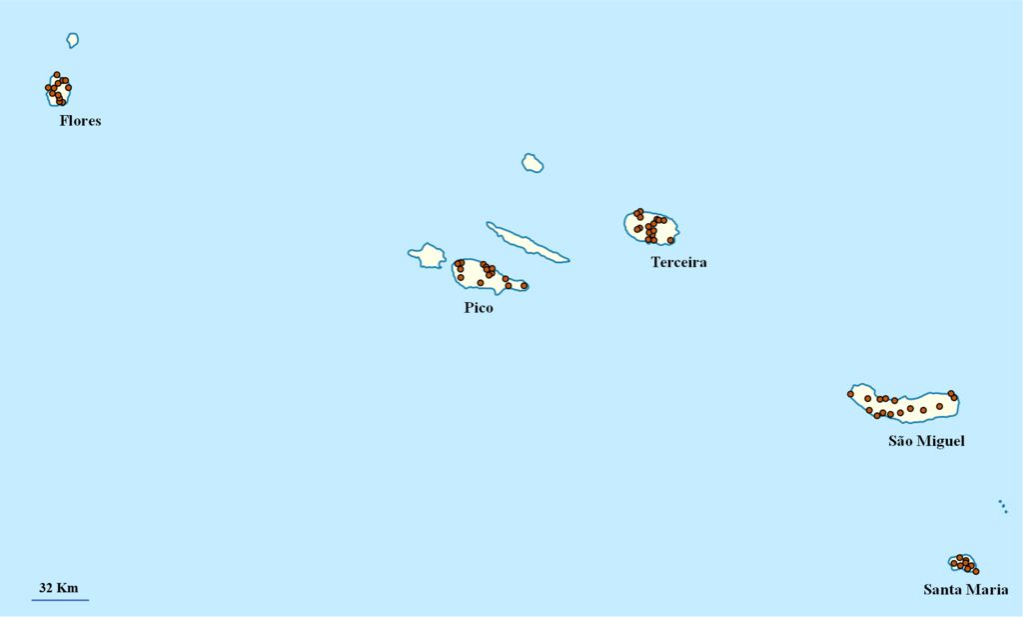
The distribution of sampling sites across the five studied Islands (n = 80).

This study intended to contribute to the current international directives concerning biodiversity, aiming to document and safeguard biological resources of the globe. Our objective was to present the most widely distributed and diverse taxa recorded during the sampling phase of the EDEN project (2008-2014) (Marcelino et al. 2013, Marcelino et al. 2020), specifically all arthropod fauna, at all strata, within eight representative habitats of five Islands of the Azores archipelago (Santa Maria, São Miguel, Terceira, Flores and Pico) (Fig. 1).

In the current study, we present an extensive checklist of Arthropoda for the following taxa: Araneae, Collembola, with emphasis on auxiliary predatory Coleoptera (Carabidae, Staphylinidae, Coccinellidae); Hymenopteran parasitoids (Aphelinidae, Bethylidae, Braconidae, Chalcididae, Chrysididae, Diapriidae, Dryinidae, Elasmidae, Encyrtidae, Eupelmidae, Figitidae, Ichneumonidae, Megaspilidae, Mymaridae, Proctotrupidae, Pteromalidae, Scelionidae, Sphecidae, Tetracampidae). These groups are particularly relevant for the following reasons:

**Arachnida: Araneae** - i) ubiquitous presence across all terrestrial habitats; ii) recognised as indicators of ecological change due to their sensitivity to cryptic changes in their habitats (Pearce and Venier 2006); iii) important predators across trophic levels (e.g. pollinators, parasitoids, saprophytes etc.), thereby impacting ecosystems’ community dynamics (Öberg and Ekbom 2006).

**Collembola** - i) the existence of a profuse diversity and abundance in a wide variety of soil systems from Islands to Continents to Antarctic habitats (Hawes et al. 2007); ii) their rapid response to changes in ecological and pedological patterns within a given ecosystem (Sousa et al. 2006).

**Hymenopteran parasitoids** - i) important role as regulators of host density (Henri and Van Veen 2011); ii) critical biological pest control agent, with circa $20 billion/year beneficial impact on US agriculture (Pennisi 2010).

**Beneficial Coleoptera** - i) the Coccinellidae groups ca. 6,000 species (Vandenberg 2002) with an ubiquitous distribution worldwide. The majority of species are predators providing relevant ecosystem and agricultural services, constituting one of the most studied groups of beneficial insects (Hodek et al. 2012, Ameixa et al. 2018 for a comprehensive revision). The rove-beetles (Staphylinidae) are one of the most diverse lineages of arthropods, inhabiting practically all terrestrial niches (Thayer 2005). They are also an ecologically-important component of soil fauna, reported to be potential bioindicators of environmental quality (Pohl et al. 2007) due to their sensitivity in detecting cryptic changes in the ecological dynamics of their ecosystems (Hodkinson and Jackson 2005)

In addition, we report widely-distributed species across sampling sites or new records for the Azores, in the orders Dermaptera, Heteroptera: Hemiptera, Neuroptera, Orthoptera, Psocoptera and Thysanoptera.

## Project description

### Title

Species inventory of Arthropoda across anthropogenically-impacted habitats in the Azores archipelago

### Personnel

Plant identifications were performed by the botanist Luis Silva, from the University of the Azores. Arthropoda sampling was performed by José A. P. Marcelino, António O. Soares, Patrícia V. Garcia and Roberto Resendes. Sorting, morphospecies IDs, image gallery stocks, digital data assembling and 96% EtOH-based collections, required a substantial number of technical staff (circa 15 people from 2010 to 2013). Species identifications were performed, initially, by José A. P. Marcelino, corroborated by Fernando Pereira and Paulo A. V. Borges using a reference collection (Dalberto Teixeira Pombo insect collection from the University of the Azores) and, subsequently, by reference taxonomists on the different Arthropoda groups, i.e. Collembola (Felipe Soto-Adames, Florida Dept. Agriculture and Consumer Services, USDA, Florida, USA), Araneae (Paulo Borges, University of the Azores), Hymenoptera (Kees van Achterberg, National History Museum Netherlands and Vladimir Žikić, University of Niš, Serbia), Coccinellidae (António O. Soares and Isabel Borges, University of the Azores), Staphylinidae (Volker Assing, Hannover, Germany) and one new record of Carabidae for the Azores by Bob Davidson at Carnegie Museum on Natural History, USA. Collembola were also genetically profiled (Marcelino et al. 2011), as well as Staphylinidae (Marcelino et al. 2016).

### Study area description

We selected the Islands, based on the relative proportion of land used in agriculture and pristine areas (based on published data by Costa et al. 2014), taking into consideration all possible combinations, i.e. São Miguel (SMG), with a high proportion of land allocated to pastureland (61%) and a low proportion of scattered native and naturalised habitats (19.1%); (ii) Terceira (TER), with high proportion of pastureland (66.9%) and a similar proportion of native and naturalised habitats as SMG, but less fragmented (21.3%); (iii) Pico (PIC), with high proportion of pastureland (50.3%) and medium/high proportion of native habitats at higher elevation (35.5%); (iv) Flores (FLO), with scarce agricultural development (17.7%) and a high proportion of native and naturalised habitats (43%); and, (v) Santa Maria (SMR), with high proportion of agricultural land (56.7%) and a low proportion of native and naturalised habitats (17.3%) (Table 1).

The importance of incorporating ecological gradients, such as an anthropogenic impact gradient, in biodiversity and conservation projects, has been previously highlighted. They constitute valuable scenarios to infer possible causes for the distribution of species across the landscape (Ulrich et al. 2009). We therefore selected habitats that represented a gradient of increasing anthropogenic impact and management intensity. Nine habitat types, divided between herbaceous and arborescent habitats, were selected to represent a comprehensive range of the flora and fauna communities. We determined consistency, prevelance and fidelity of a given plant species across all habitats to define them, based on their flora. We used a metric called IndVal and developed another called SiteVal which can now be used to assign a location to a given habitat (and anthropogenic level of influence) (see more details in Marcelino et al. 2013, Marcelino et al. 2014, Silva et al. 2017). The herbaceous habitat gradient (Table 2) ranged from natural meadows (MED) to corn fields (COR). The arborescent habitat gradient (Table 2) ranged from natural pristine forests of *Laurus azorica* (NAT) to orchards of *Citrus* sp. (ORC). Pristine meadows were not present on Santa Maria and Terceira and semi-natural pastures at low altitude (SNPL) were used as a surrogate for MED on these Islands.

### Design description

In order to obtain the maximum information on arthropod biodiversity, all strata present at a given habitat type were sampled, i.e. micro-epigean fauna (Berlese-Tullgren trapping), soil fauna (Pitfall trapping), aerial vagility fauna (Suction with an aspirator) and canopy fauna (sweeping nets).

One Island per week was sampled during the summer 2009 (July-August). This eliminated seasonal effects in the sampling. The total number of samples was 4800 [80 sampling sites x 4 different types of traps x 15 samples per site].

The samples were subsequently processed in laboratory facilities and assigned to morphospecies groups, progressing to higher taxonomic degrees of identifications. Species richness and abundance were recorded. Species accumulation curves were performed for inventory completeness using EstimateS (using the ratio of observed to the estimated species richness with the non-parametric estimator Jackknife) (Colwell 2013). Inventory completeness was 70-75% for Staphylinidae and Collembola (Marcelino et al. 2011, Marcelino et al. 2016), reaching 80% for Araneae and Hymenoptera parasitoids (data not published).

### Funding

This study was financially supported by FLAD – Fundação Luso-Americana para o Desenvolvimento and by the Direção Regional da Ciência e Tecnologia (DRCT) & PROEMPREGO, of the Azores. This study was also financed by FEDER in 85% and by Azorean Public funds by 15% through Operational Programme Azores 2020, under the following projects AZORESBIOPORTAL –PORBIOTA (ACORES-01-0145-FEDER-000072), and under the project ECO^2^-TUTA (ACORES-01-0145-FEDER-000081).

## Sampling methods

### Study extent

Five Islands of the Azores: Santa Maria, São Miguel, Terceira, Pico and Flores.

### Sampling description

Suction (SU), sweeping (SW) and soil (BT) sampling were obtained in parallel with the pitfall traps (PF) in the sites previously listed (Table 1), in equal numbers of samples and distance.

SU and SW were done to record species at strata other than the epigeic stratum. SU was made with a hand-held aspirator (Stihl BG55), collecting the arthropods in shrubs, when available. SU was made individually for ca. 8 seconds, at each of four exposures (i.e. N, S, W, E) of the shrub or agro-culture plant. The specimens from all four cardinal exposures were transferred to a single cup for each SU and SW sampling spot, respectively. SW was made by gently sweeping with a 64 cm diameter sweeping net.

Berlese-Tullgreen sampling (BT) was made by collecting ca. 100 grams of soil litter per sampling unit (15 samples for each transect established at PF sampling spots). Samples were immediately stored in a cooler to avoid proliferation of saprophytic fungi and sent by air transportation to the Department of Biology, University of the Azores, Ponta Delgada where they were placed in a cooling chamber at 4°C for subsequent processing in BT traps. BT trap units consisted of two plastic darkened containers, assembled together to provide an upper vented area (14 cm diameter x 11.5 cm high) with 4 openings (1 cm diameter covered with a 0.3 x 0.3 mm diameter mesh) and coupled with a 15 W lamp on top. The lower collecting area (13 cm diameter x 10 cm high) was partially filled with ca. 80 ml of the same mixture used in PF. Litter samples were placed on a 1.8 x 1.8 mm mesh, attached to a plastic funnel positioned in the assembling zone between the two halves of the device. In order to avoid heat and dryness, Collembola and other micro-arthropods crawled downwards to the littler sample and dropped through the funnel into the collecting mixture. Litter samples remained for 72 h in BT before specimen sorting at laboratory facilities.

Two parallel transects with fifteen pitfall traps (PF) were placed in 150 x 150 m geo-referenced plots. PF consisted of plastic cylinder cups 78 mm deep and 42 mm diameter filled with ca. 80 ml of a mixture of 96% Ethanol and 0.05% liquid detergent. PFs were buried in the soil so that the lid was flush with the surface and covered with a plastic plate at ca. 3 cm height, to avoid desiccation, flooding or insectivore predation. Traps remained in the soil for 7 days prior to collection. For each habitat type and Island, two replicate sites were monitored (with a minimum distance of 5 km apart), for a total of 80 sampling sites (i.e. 5 Islands x 8 habitat types per Island x 2 sites for each habitat type).

All specimens where stored in 96% EtOH in order to maintain viability for future genetic and/or taxonomic work, as well as voucher exchanges with other institutions.

### Quality control

Identifications were conducted in a progressive higher degree of taxonomy resolution, i.e. 1) morphospecies were generated and, concomitantly, an ongoing web-based image gallery stock was created (at www.eden-azores.webs.com). This secured consistency in assigning specimens to morpho-species without duplications; 2) voucher specimens of all morphospecies were taxonomically assigned to a genus and, if possible, to species level; 3) species of Collembola and Staphylinidae were genetically profiled to match genetic and morphological IDs; 4) All voucher specimens where sent to taxonomist referees in the respective Order, family, genus or group (taxonomists listed in the Personnel section of this report), which corroborated identifications from steps 1, 2 and 3.

## Geographic coverage

### Description

Azores (Portugal). Islands of Santa Maria, São Miguel, Terceira, Pico and Flores

### Coordinates

36.906 and 39.589 Latitude; -31.311 and -24.961 Longitude.

## Taxonomic coverage

### Description

Widely distributed Arthropoda groups are reported, i.e. Araneae, Collembola, Hymenopteran parasitoids and beneficial Coleoptera (e.g. Carabidae, Coccinellidae, Staphylinidae). In addition, Dermaptera, Hemiptera, Neuroptera, Orthoptera, Psocoptera and Thysanoptera. Information can be retrieved in the data resources below (Table of Species Occurrence).

### Taxa included

**Table:** 

Rank	Scientific Name	Common Name
order	Araneae	Spiders
order	Collembola	Springtails
kingdom	Dermaptera	Earwigs
order	Orthoptera	Grasshopper
order	Thysanoptera	Thrips
kingdom	Hemiptera	Tru bugs
order	Psocoptera	Booklice
order	Coleoptera	Beetles
family	Neuroptera	Net-winged insects (lacewings)
order	Hymenoptera	Wasps, ants

## Temporal coverage

### Notes

Vegetation landcover and Arthropoda biodiversity were sampled during the summer of 2009 (July-August).

## Collection data

### Collection name

Arthropoteca of the University of the Azores at Ponta Delgada, São Miguel

### Collection identifier

EDEN Azores

### Specimen preservation method

All specimens were preserved in 96% ethanol.

### Curatorial unit

EDEN Azores Arthropoteca at the University of the Azores

## Usage licence

### Usage licence

Creative Commons Public Domain Waiver (CC-Zero)

## Data resources

### Data package title

EDEN Arthropod Azores Database

### Resource link

https://www.gbif.org/dataset/5cc85d78-4313-4959-b17d-cd3dc32cc155

### Alternative identifiers

http://ipt.gbif.pt/ipt/resource?r=eden_arthropod_database_azores

### Number of data sets

1

### Data set 1.

#### Data set name

EDEN Arthropod Azores Database

#### Data format

Darwin Core Archive

#### Number of columns

53

#### Download URL

http://ipt.gbif.pt/ipt/resource?r=eden_arthropod_database_azores

#### Data format version

Version 1

#### Description

The following data table includes all the records for which a taxonomic identification of the species was possible. The dataset submitted to GBIF is structured as a sample event dataset, with two tables: event (as core) and occurrences. The data in this sampling event resource have been published as a Darwin Core Archive (DwCA), which is a standardised format for sharing biodiversity data as a set of one or more data tables. The core data file contains 3214 records (eventID) and the occurrences file 19555 records (occurrenceID). This IPT archives the data and thus serves as the data repository. The data and resource metadata are available for download from Marcelino et al. (2020).

**Data set 1.** 

Column label	Column description
Table of Sampling Events	Table with sampling events data (beginning of table)
eventID	Identifier of the events, unique for the dataset
stateProvince	Name of the region of the sampling site
islandGroup	Name of archipelago
island	Name of the Island
country	Country of the sampling site
countryCode	ISO code of the country of the sampling site
locationRemarks	Details on the locality site
decimalLatitude	Approximate centre point decimal latitude of the field site in GPS coordinates
decimalLongitude	Approximate centre point decimal longitude of the field site in GPS coordinates
geodeticDatum	The ellipsoid, geodetic datum or spatial reference system (SRS) upon which the geographic coordinates given in decimalLatitude and decimalLongitude are based
coordinateUncertaintyInMetres	Uncertainty of the coordinates of the centre of the sampling plot
coordinatePrecision	A decimal representation of the precision of the coordinates given in the decimalLatitude and decimalLongitude
georeferenceSources	A list (concatenated and separated) of maps, gazetteers or other resources used to georeference the Location, described specifically enough to allow anyone in the future to use the same resources
verbatimElevation	Elevation in metres
fieldNumber	With a code for the sample
samplingProtocol	The sampling protocol used to capture the species
eventDate	Date or date range the record was collected
year	Year of the event
month	Month of the event
day	Day of the event
habitat	The surveyed habitat
eventRemarks	Comments or notes about the type of habitat
locationID	Identifier of the location
Table of Species Occurrence	Table with species abundance data
type	Type of the record, as defined by the Public Core standard
licence	Reference to the licence under which the record is published
institutionID	The identity of the institution publishing the data
collectionID	The identity of the collection publishing the data
institutionCode	The code of the institution publishing the data
collectionCode	The code of the collection where the specimens are conserved
datasetName	Name of the dataset
basisOfRecord	The nature of the data record
occurrenceID	Identifier of the record, coded as a global unique identifier
catalogNumber	Record number of the specimen in the collection
recordedBy	Name of the person who performed the sampling of the specimens
individualCount	Total number of individuals captured
establishmentMeans	The process of establishment of the species in the location, using a controlled vocabulary: 'native', 'introduced', 'endemic'
eventID	Identifier of the events, unique for the dataset
identifiedBy	The names of taxonomists who assigned the Taxon to the subject
dateIdentified	The date on which the subject was determined as representing the taxon
identificationRemarks	Comments or notes about the Identification
scientificName	Complete scientific name including author and year
kingdom	Kingdom name
phylum	Phylum name
class	Class name
order	Order name
family	Family name
genus	Genus name
specificEpithet	Specific epithet
infraspecificEpithet	Subspecies name
taxonRank	Lowest taxonomic rank of the record
scientificNameAuthorship	Name of the author of the lowest taxon rank included in the record

## Additional information

We collected a total of 116,523 specimens belonging to 483 species and subspecies of selected groups of arthropods. Due to the unavailability of taxonomic expertise, these represent a sub-set of the Arthropoda recorded during the monitoring programme EDEN (2008-2014) carried out in the Azores archipelago. Hymenoptera, mostly parasitoids (193 species and mophospecies), Coleoptera (95 species); Collembola (89 species); and Araneae (72 species) are the most represented taxa (Table 3). A total of 28 species are endemic to the Azores archipelago (2511 specimens), 59 are native non-endemic (26,139 specimens) and 161 are introduced (54,601 specimens). For 238 taxa identified as morphospecies (mostly Collembola and Hymenoptera), the colonisation status is unknown (33,272 specimens) (see Table 3).

The ten most abundant species account for 54% of all the sampled specimens. These ten species include only two native non-endemic taxa, the ant *Lasius grandis* Forel, 1990 (that ranks as second) and the aphid *Cinara juniperi* (De Geer, 1773) (that ranks as fifth), a specialist species associated with the Azorean endemic tree *Juniperus bervifolia*. All the other dominant species include six introduced species and two morphospecies of unknown status (see Table 3).

The most relevant data reported in this study are the new non-native species for the Azores, i.e. two beetles (Coleoptera), one Hemiptera-Heteroptera: Pentatomidae, six Collembola and 29 Hymenoptera micro-parasitoids (see Table 3 and Borges et al. 2010). These 37 taxa were found in several Islands (see below) and correspond to the addition of five new species for Flores Island, 10 species for Pico Island, 12 species for Terceira Island, 19 species for S. Miguel Island and five species for S. Maria Island.

Additional species records for the Islands included: Flores (5 Collembola; 9 Araneae; 2 Hemiptera; 8 Coleoptera; 8 Hymenoptera), Pico (4 Collembola; 7 Araneae; 4 Hemiptera; 11 Coleoptera; 9 Hymenoptera), Terceira (4 Collembola; 1 Araneae; 3 Hymenoptera), S. Miguel (1 Araneae; 2 Coleoptera; 3 Hymenoptera), S. Maria (5 Collembola; 3 Araneae; 2 Hemiptera; 2 Hymenoptera) (see Table 3).

The two species of beetles include a ground-beetle *Asaphidion flavipes* (Linnaeus, 1761) (Carabidae) found in an exotic forest (*Eucalyptus* spp. plantation) in S. Miguel Island and the rove-beetle *Tachyporus dispar* (Paykull, 1789) (Staphylinidae), found in Flores and S. Miguel Islands, in pasture land and also in *Cryptomeria japonica* plantations.

The bug *Acrosternum heegeri* Fieber, 1861 (Pentatomidae) was found in corn fields, pastures and orchards in the Islands of Flores and Terceira.

Concerning the Collembola, the new species, recorded to Azores, include: *Entomobrya regularis* Stach, 1963 (Entomobryidae) found in *Cryptomeria japonica* plantations from S. Miguel Island; *Lepidocyrtus lusitanicus piezoensis* (Simón-Benito, 2007) (Entomobryidae) found in pastures, corn fields and exotic forests in three Islands (Terceira, S. Miguel and S. Maria); *Sminthurinus quadrimaculatus* (Ryder, 1879) (Katiannidae) found in native forest and *Cryptomeria japonica* plantations in Terceira and S. Maria Islands and *Jordanathrix articulata* (Ellis, 1974) (Sminthuridae) found in all the studied Islands and in almost all sampled habitats, this being a very common species.

Two Collembola genera are also new records for Azores: *Himalanura* Baijal, 1958 (Entomobryidae) and *Protophorura* Absolon, 1901 (Onychiuridae). Further taxonomic resolution is needed to confirm their status and species assignation.

Concerning the Hymenoptera parasitoids, several species and genera are also new records for the Azores:

*Gonatopus clavipes* (Thunberg, 1827) (Dryinidae), sampled in corn fields in S. Miguel Island.

*Aphidius colemani* Viereck, 1912 (Braconidae), sampled in pastures, corn fields and native forest in Pico, Terceira and S. Miguel Islands.

*Aphidius ervi* Haliday, 1834 (Braconidae), sampled in pastures and corn fields in Pico and Terceira Islands.

*Aphidius matricariae* Viereck, 1912 (Braconidae), sampled in pastures and native forest in Pico and Terceira Islands.

*Aphidius rhopalosiphi* Stefani-Perez, 1902 (Braconidae), sampled in all types of pastures (i.e. low and high altitude), corn fields, orchards and native forest in Pico, Terceira, S. Miguel and S. Maria Islands.

*Aphidius rosae* (Haliday, 1834) (Braconidae), sampled in semi-natural pastures in Pico Island.

*Aphidius urticae* Haliday, 1834 (Braconidae), sampled in all types of pastures and corn fields, in Flores, Pico, Terceira and S. Miguel Islands.

*Centistidea ectoedemiae* Rohwer, 1914 (Braconidae), sampled in pastures, orchards and *Cryptomeria japonica* plantations, in Flores, Pico, Terceira and S. Maria Islands.

*Meteorus unicolor* (Wesmael, 1835) (Braconidae), sampled in all types of pastures, orchards, exotic forest and native forest in Flores, Pico and Terceira Islands.

*Meteorus collaris* (Spin.) Hal. – Ruschka, Fulmek, 1915 (Braconidae), sampled in corn fields and exotic forest in Pico and Terceira Islands.

*Orthostigma cratospilum* (Thomson, 1895) (Braconidae), sampled in pastures, orchards, exotic forest and native forest in Pico, Terceira and S. Miguel Islands.

*Orthostigma latriventris* Ratzeburg, 1844 (Braconidae), sampled in pastures, corn fields and orchards in Pico, Terceira and S. Maria Islands. Two other morphospecies of *Orthostigma* sp. are recorded occurring in all the Islands and habitats, but for which further taxonomic resolution is needed to confirm their status and species assignation.

*Pseudopezomachus bituberculatus* (Marshall, 1905) (Braconidae), sampled in semi-natural pastures in Pico and S. Miguel Islands.

*Tanycarpa punctata* (van Achterberg, 1976) (Braconidae), sampled in *Cryptomeria japonica* plantations, on S. Miguel Island.

New genera, not previously recorded in the Azores, include: *Pycnetron* sp. (Chalcidoidea: Pteromalidae); four species of *Aspilota* sp. (Braconidae: Aphidiinae); four species of *Chorebus* sp. (Braconidae: Alysiinae); *Microgaster* sp. (Braconidae: Microgastrinae); *Homolobus* sp. (Braconidae: Homolobinae); *Lodbrokia* sp. (Braconidae: Alysiinae). Further taxonomic resolution is needed to confirm their status and species assignation.

We wish also to call attention to three species collected in our study for the first time in Azores, but reported in previous publications: two spiders species of the family (Phrurolithidae): *Liophrurillus flavitarsis* (Lucas, 1846) found in an exotic forest in S. Maria Island and *Phrurolinillus lisboensis* Wunderlich, 1995 found in pastures in Pico and Terceira Islands (see Borges et al. 2013); the ladybird *Nephus voeltzkowi* (Weise, 1910) (Coccinellidae) found in orchards and semi-natural pastures in Terceira Island (see Magro et al. 2020).

The staggering number of Hymenoptera, mostly parasitoids (193 species and morphospecies) concurs with reports of an increasingly unreported high number of species in this group, which, due to their size, makes capture and identification difficult and, therefore, underestimated (Lobo and Borges 2010). Their number might rival Coleoptera, commonly reported as the most speciose animals on Earth (Zhang et al. 2018). The number of Hymenoptera parasitoid species is thought to be 2.5-3.2-fold higher than the one of Coleoptera species (Forbes et al. 2018). We hypothesise that the same pattern for Collembola exists as identifications, based on morphological characters, is usually insufficient to discriminate phenotypic identical species. This was the case for Collembola (Marcelino et al. 2011) and Staphylinidae (Marcelino et al. 2016), in which, after matching morphological identifications with genetic profiles, undetected cryptic species complexes were found.

Our results indicate that increasing anthropogenic impact is a major driver for species diversity in habitats ranging from pristine to highly human-influenced habitats. Our results support the mission statement of Borges et al. (2018) that there is the urgent need to inventory and monitor island biodiversiy.

## Figures and Tables

**Table 1.** Sampled Sites (n = 80).

Island	Habitat	Locality	Elevation (m)	Latitude	Longitude
Flores	Corn Crop	Fajã Grande	64	39.4572	-31.2611
Flores	Corn Crop	Ponta Delgada	34	39.5187	-31.2102
Flores	Invasive Forest	Monte	190	39.4556	-31.1441
Flores	Invasive Forest	Saída das Lajes	256	39.3879	-31.1954
Flores	Meadows	Estrada para o Morro Alto	683	39.4536	-31.2354
Flores	Meadows	Zona das Lagoas / Fundão	697	39.4079	-31.1977
Flores	Natural Forest	Estrada para Ponta Delgada	729	39.4739	-31.2089
Flores	Natural Forest	Estrada para Ponta Delgada / Lixeira	579	39.4882	-31.1858
Flores	Orchard	Lajes (SDA)	109	39.3864	-31.1672
Flores	Orchard	Vales	186	39.4522	-31.1464
Flores	Pasture	Cedros	344	39.4808	-31.1594
Flores	Pasture	Lajes	205	39.3860	-31.1842
Flores	Production forest	Lajes Saída 1	270	39.3914	-31.2012
Flores	Production forest	Ponta Ruiva	494	39.4872	-31.1778
Flores	Semi-natural Pasture	Estrada para as Fajãs	502	39.4322	-31.2339
Flores	Semi-natural Pasture	Leste do Morro Alto	619	39.4178	-31.2013
Pico	Corn Crop	Madalena (Sete Cidades)	90	38.5337	-28.5097
Pico	Corn Crop	Piedade	115	38.4216	-28.0513
Pico	Invasive Forest	Cabeço do Chão	443	38.5424	-28.4829
Pico	Invasive Forest	São Roque	767	38.5170	-28.3170
Pico	Meadows	Encosta da Prainha 2	797	38.4406	-28.1866
Pico	Meadows	Meia encosta da Prainha	765	38.4422	-28.1919
Pico	Natural Forest	Estrada para as Lagoas	715	38.4674	-28.2967
Pico	Natural Forest	Mistério da Prainha	675	38.4822	-28.2610
Pico	Orchard	Madalena (Sete Cidades)	79	38.5337	-28.5097
Pico	Orchard	Prainha	41	38.4686	-28.2025
Pico	Pasture	Criação Velha	214	38.5090	-28.4914
Pico	Pasture	S. Miguel Arcanjo	150	38.5036	-28.2952
Pico	Production forest	Candelária	258	38.4705	-28.4929
Pico	Production forest	Prédio Mário Sequeira	419	38.4933	-28.2952
Pico	Semi-natural Pasture	Corre Água	708	38.4768	-28.2975
Pico	Semi-natural Pasture	Longitudinal (Km 5)	896	38.4454	-28.3628
Santa Maria	Corn Crop	Água de Alto / Caminho Florestal do Alto	227	36.9925	-25.1072
Santa Maria	Corn Crop	Santa Bárbara	201	36.9915	-25.0698
Santa Maria	Invasive Forest	Anjos	166	36.9922	-25.1415
Santa Maria	Invasive Forest	Estrada para Santo Espírito	236	36.9590	-25.0667
Santa Maria	Natural Forest	Estrada para o Pico Alto	491	36.9758	-25.0866
Santa Maria	Natural Forest	Pico Alto	580	36.9826	-25.0909
Santa Maria	Orchard	Malbusca	260	36.9471	-25.0716
Santa Maria	Orchard	Trevina	218	36.9874	-25.1096
Santa Maria	Pasture	Calheta, Saída da Maia	266	36.9436	-25.0302
Santa Maria	Pasture	Estrada para Santa Bárbara / Forno	354	36.9661	-25.0584
Santa Maria	Production forest	Almagreira	198	36.9715	-25.1252
Santa Maria	Production forest	Fontinhas / Parque Florestal	238	36.9595	-25.0721
Santa Maria	Semi-natural Pasture	Arrebetão	453	36.9738	-25.0782
Santa Maria	Semi-natural Pasture	Loural / Estrada	428	36.9596	-25.0691
Santa Maria	Semi-natural Pasture Low Altitude	Aeroporto (100m)	176	36.9715	-25.1622
Santa Maria	Semi-natural Pasture Low Altitude	Paúl / Estrada para o Anjo	198	36.9918	-25.1392
São Miguel	Corn Crop	Contentores da Unileite	38	37.7415	-25.6850
São Miguel	Corn Crop	Rabo de Peixe	34	37.8138	-25.5479
São Miguel	Invasive Forest	Pico das Camarinhas	175	37.8583	-25.8484
São Miguel	Invasive Forest	S. Pedro	378	37.8358	-25.1826
São Miguel	Meadows	Encosta Sul para a Lagoa do Fogo	745	37.7650	-25.5024
São Miguel	Meadows	Próximo do Monte Escuro / vertente virada a norte	824	37.7827	-25.4532
São Miguel	Natural Forest	Abelheira	490	37.7590	-25.6416
São Miguel	Natural Forest	Tronqueira - Miradouro	627	37.7874	-25.2841
São Miguel	Orchard	Aflitos	104	37.8126	-25.6396
São Miguel	Orchard	Malaca da Lagoa	203	37.7583	-25.5787
São Miguel	Pasture	Calhetas, Rabo de Peixe	30	37.8213	-25.6059
São Miguel	Pasture	Rocha da Relva	101	37.7631	-25.7345
São Miguel	Production forest	Lagoa das Empadadas	775	37.8265	-25.7506
São Miguel	Production forest	Sto António	190	37.8510	-25.2013
São Miguel	Semi-natural Pasture	Altiprado	640	37.7706	-25.3887
São Miguel	Semi-natural Pasture	Lagoa do Fogo / Vertente Sul	660	37.7658	-25.5100
Terceira	Corn Crop	Etar	51	38.6543	-27.2006
Terceira	Corn Crop	S. Brás	159	38.7606	-27.1367
Terceira	Invasive Forest	Biscoitos/ Estrada para Sul	315	38.7716	-27.2636
Terceira	Invasive Forest	Estrada para a Agualva	409	38.7639	-27.1923
Terceira	Natural Forest	Caldeira Guilherme Muniz	537	38.7060	-27.2032
Terceira	Natural Forest	Algar do Carvão -Terra Brava	621	38.7399	-27.2047
Terceira	Orchard	Biscoitos	125	38.7882	-27.2737
Terceira	Orchard	Terra Chã	72	38.6641	-27.2524
Terceira	Pasture	Agualva	234	38.7608	-27.1651
Terceira	Pasture	Estrada de Porto Santo/ Angra	294	38.6789	-27.2205
Terceira	Production forest	Lagoa das Papas	545	38.7161	-27.2894
Terceira	Production forest	Posto Santo/ Fim estrada	412	38.7003	-27.2423
Terceira	Semi-natural Pasture	Furna do Enxofre	554	38.7268	-27.2322
Terceira	Semi-natural Pasture	Pico Gaspar	461	38.7305	-27.2743
Terceira	Semi-natural Pasture Low Altitude	Biscoitos	14	38.8000	-27.2505
Terceira	Semi-natural Pasture Low Altitude	Porto Martins	5	38.6773	-27.0622

**Table 2.** Increasing gradients of anthropogenic influence in herbaceous communities (1-5) and arborescent communities (1-4). Description and characteristic plant species communities. Note: * - Semi-natural pastures, at low altitude (SNPL), replaced Meadow habitats (MED) in Santa Maria and Terceira Islands due to the lack of sampling sites of the latter community type in these Islands.

**Gradient of HERBACEOUS communities**	**Composition**	**Plant species communities**
1. Natural meadows (MED)	Indigenous taxa. Low management intensity and anthropogenic influence	*Holcus rigidus*, *Festuca* spp., *Deschampsia foliosa*, *Leontodon* spp., *Tolpis azorica*
2. Semi-natural pastures at low altitude (SNPL)*	Annual populations of *Daucus carota*. Low management intensity and anthropogenic impact	*Sporobolus indicus*, *Briza minor*, *Lotus subbiflorus*
3. Semi-natural pastures at high altitude (SNP)	Non-indigenous taxa with seldom indigenous taxa. Low management intensity and anthropogenic influence	*Holcus lanatus*, *Agrostis castellana*, *Polytrichum commune*, *Ranunculus repens*, *Juncus effusus*, *Selaginella kraussiana*, *Sibthorpia europeia*, *Eleocharis multicaulis*, *Sherardia arvensis*, *Anagallis arvensis*
4. Artificial pastures (PAS)	Introduced taxa. High human-management intensity and anthropogenic influence	*Lolium perenne*, *Bromus willdenowii*, *Trifolium repens*, *Poa* spp., *Holcus lanatus*, *Rumex obtusifolius*, *Plantago lanceolata*, *Dactylis glomerata*, *Sporobolus indicus*
5. Crops (COR)	Introduced taxa. High human-management intensity, high anthropogenic influence, as well as pesticide and fertiliser use	*Zea mays*, weeds and ruderal plants
**Gradient of ARBORESCENT communities**	**Composition**	**Main species**
1. Natural Forests (NAT)	Indigenous taxa. Low management intensity and anthropogenic influence	*Laurus azorica*, *Juniperus brevifolia*, *Erica azorica*, Ilex perado subsp. azorica, *Morella faya*
2. Exotic forests (INV)	Non-indigenous invasive taxa. Low to medium management intensity and anthropogenic influence	*Pittosporum undulatum*, *Acacia melanoxylon*, *Eucalyptus globulus*, *Pinus pinaster*, *Solanum mauritianum*
3. Production Forest (PRO)	Human intentionally introduced taxa. High management intensity and anthropogenic influence	*Cryptomeria japonica* (monocultural stands)
4. Orchards (ORC)	Introduced taxa. Medium management intensity and anthropogenic influence	*Citrus sinensis*, *Mallus domestica*, *Prunus* spp., other crops, weeds, ornamentals and rudereal species

**Table 3.** List of species and morphospecies with information on the colonisation status (CS) and abundance (n). The taxa are listed following the alphabetical sequence of classes, orders within classes, families within orders and finally species within families. When a species is a new record for a given Island, we add that information (codes for Islands as follows: FLO - Flores; PIC - Pico; TER - Terceira; SMG - S. Miguel; SMR - S. Maria). The top ten most abundant species are marked with an *.

Class	Order	Family	Species	CS	n
Arachnida	Araneae	Agelenidae	*Lycosoides coarctata* (Dufour, 1831)	Introduced	9
Arachnida	Araneae	Agelenidae	*Malthonica* sp.	Introduced	3
Arachnida	Araneae	Agelenidae	*Tegenaria domestica* (Clerck, 1757)	Introduced (New to PIC)	7
Arachnida	Araneae	Agelenidae	*Textrix caudata* L. Koch, 1872	Introduced	3
Arachnida	Araneae	Araneidae	*Gibbaranea occidentalis* Wunderlich, 1989	Endemic	45
Arachnida	Araneae	Araneidae	*Mangora acalypha* (Walckenaer, 1802)	Introduced	12
Arachnida	Araneae	Cheiracanthiidae	*Cheiracanthium erraticum* (Walckenaer, 1802)	Introduced	1
Arachnida	Araneae	Cheiracanthiidae	*Cheiracanthium floresense* Wunderlich, 2008	Endemic	4
Arachnida	Araneae	Clubionidae	*Clubiona terrestris* Westring, 1851	Introduced	87
Arachnida	Araneae	Clubionidae	*Porrhoclubiona decora* (Blackwall, 1859)	Native	5
Arachnida	Araneae	Clubionidae	*Porrhoclubiona genevensis* (L. Koch, 1866)	Introduced (New to FLO, SMG)	90
Arachnida	Araneae	Dictynidae	*Emblyna acoreensis* Wunderlich, 1992	Endemic (New to SMG, SMR)	40
Arachnida	Araneae	Dictynidae	*Lathys dentichelis* (Simon, 1883)	Native	133
Arachnida	Araneae	Dictynidae	*Nigma puella* (Simon, 1870)	Introduced	59
Arachnida	Araneae	Dysderidae	*Dysdera crocata* C.L. Koch, 1838	Introduced	168
Arachnida	Araneae	Gnaphosidae	*Haplodrassus signifer* (C.L. Koch, 1839)	Introduced	19
Arachnida	Araneae	Gnaphosidae	*Marinarozelotes lyonneti* (Audouin, 1826)	Introduced (New to FLO, PIC)	9
Arachnida	Araneae	Gnaphosidae	*Zelotes tenuis* (L. Koch, 1866)	Introduced (New to FLO, PIC)	24
Arachnida	Araneae	Linyphiidae	*Agyneta depigmentata* Wunderlich, 2008	Endemic	12
Arachnida	Araneae	Linyphiidae	*Agyneta fuscipalpa* (C.L. Koch, 1836)	Introduced	642
Arachnida	Araneae	Linyphiidae	*Agyneta* sp.		10
Arachnida	Araneae	Linyphiidae	*Canariphantes acoreensis* (Wunderlich, 1992)	Endemic	27
Arachnida	Araneae	Linyphiidae	*Entelecara schmitzi* Kulczynski, 1905	Native	11
Arachnida	Araneae	Linyphiidae	*Erigone atra* Blackwall, 1833	Introduced	348
Arachnida	Araneae	Linyphiidae	*Erigone autumnalis* Emerton, 1882	Introduced	882
Arachnida	Araneae	Linyphiidae	*Erigone dentipalpis* (Wider, 1834)	Introduced	257
Arachnida	Araneae	Linyphiidae	*Erigone* sp.		7
Arachnida	Araneae	Linyphiidae	*Mermessus bryantae* (Ivie & Barrows, 1935)	Introduced (New to FLO)	49
Arachnida	Araneae	Linyphiidae	*Mermessus fradeorum* (Berland, 1932)	Introduced	190
Arachnida	Araneae	Linyphiidae	*Microctenonyx subitaneus* (O. Pickard-Cambridge, 1875)	Introduced	1
Arachnida	Araneae	Linyphiidae	*Microlinyphia johnsoni* (Blackwall, 1859)	Native	33
Arachnida	Araneae	Linyphiidae	*Minicia floresensis* Wunderlich, 1992	Endemic (New to SMR)	8
Arachnida	Araneae	Linyphiidae	*Neriene clathrata* (Sundevall, 1830)	Introduced	3
Arachnida	Araneae	Linyphiidae	*Oedothorax fuscus* (Blackwall, 1834)	Introduced *	1991
Arachnida	Araneae	Linyphiidae	*Ostearius melanopygius* (O. Pickard-Cambridge, 1879)	Introduced	129
Arachnida	Araneae	Linyphiidae	*Palliduphantes schmitzi* (Kulczynski, 1899)	Native	174
Arachnida	Araneae	Linyphiidae	*Pelecopsis parallela* (Wider, 1834)	Introduced	74
Arachnida	Araneae	Linyphiidae	*Prinerigone vagans* (Audouin, 1826)	Introduced	221
Arachnida	Araneae	Linyphiidae	*Savigniorrhipis acoreensis* Wunderlich, 1992	Endemic	467
Arachnida	Araneae	Linyphiidae	*Tenuiphantes miguelensis* (Wunderlich, 1992)	Native	417
Arachnida	Araneae	Linyphiidae	*Tenuiphantes tenuis* (Blackwall, 1852)	Introduced	1234
Arachnida	Araneae	Linyphiidae	*Walckenaeria grandis* (Wunderlich, 1992)	Endemic	4
Arachnida	Araneae	Lycosidae	*Arctosa perita* (Latreille, 1799)	Introduced (new PIC, SMR)	27
Arachnida	Araneae	Lycosidae	*Pardosa acorensis* Simon, 1883	Endemic	499
Arachnida	Araneae	Mimetidae	*Ero furcata* (Villers, 1789)	Introduced	3
Arachnida	Araneae	Nesticidae	*Eidmannella pallida* (Emerton, 1875)	Introduced	1
Arachnida	Araneae	Oecobiidae	*Oecobius similis* Kulczynski, 1909	Native (New to PIC)	11
Arachnida	Araneae	Phrurolithidae	*Liophrurillus flavitarsis* (Lucas, 1846)	Introduced	5
Arachnida	Araneae	Phrurolithidae	*Phrurolinillus lisboensis* Wunderlich, 1995	Introduced	4
Arachnida	Araneae	Pisauridae	*Pisaura acoreensis* Wunderlich, 1992	Endemic	2
Arachnida	Araneae	Salticidae	*Chalcoscirtus infimus* (Simon, 1868)	Introduced	9
Arachnida	Araneae	Salticidae	*Heliophanus kochii* Simon, 1868	Introduced	15
Arachnida	Araneae	Salticidae	*Macaroeris cata* (Blackwall, 1867)	Native	61
Arachnida	Araneae	Salticidae	*Macaroeris diligens* (Blackwall, 1867)	Native (New to FLO)	13
Arachnida	Araneae	Salticidae	*Neon acoreensis* Wunderlich, 2008	Endemic	6
Arachnida	Araneae	Salticidae	*Pseudeuophrys vafra* (Blackwall, 1867)	Introduced	12
Arachnida	Araneae	Salticidae	*Salticus mutabilis* Lucas, 1846	Introduced (New to PIC)	29
Arachnida	Araneae	Salticidae	*Synageles venator* (Lucas, 1836)	Introduced	2
Arachnida	Araneae	Tetragnathidae	*Metellina merianae* (Scopoli, 1763)	Introduced	17
Arachnida	Araneae	Tetragnathidae	*Pachygnatha degeeri* Sundevall, 1830	Introduced (New to FLO, PIC)	31
Arachnida	Araneae	Tetragnathidae	*Sancus acoreensis* (Wunderlich, 1992)	Endemic	7
Arachnida	Araneae	Theridiidae	*Cryptachaea blattea* (Urquhart, 1886)	Introduced	7
Arachnida	Araneae	Theridiidae	*Lasaeola oceanica* Simon, 1883	Endemic	90
Arachnida	Araneae	Theridiidae	*Neottiura bimaculata* (Linnaeus, 1767)	Introduced (New to FLO)	14
Arachnida	Araneae	Theridiidae	*Rugathodes acoreensis* Wunderlich, 1992	Endemic	72
Arachnida	Araneae	Theridiidae	*Steatoda grossa* (C.L. Koch, 1838)	Introduced	63
Arachnida	Araneae	Theridiidae	*Theridion melanurum* Hahn, 1831	Introduced (New to FLO, TER)	19
Arachnida	Araneae	Theridiidae	*Theridion musivivum* Schmidt, 1956	Native	5
Arachnida	Araneae	Thomisidae	*Xysticus cor* Canestrini, 1873	Native	26
Arachnida	Araneae	Thomisidae	*Xysticus nubilus* Simon, 1875	Introduced	69
Arachnida	Araneae	Zodariidae	*Zodarion atlanticum* Pekár & Cardoso, 2005	Introduced (New to FLO)	130
Arachnida	Araneae	Zoropsidae	*Zoropsis spinimana* (Dufour, 1820)	Introduced (New to PIC)	1
Entognatha	Collembola	Entomobryidae	*Entomobrya albocincta* (Templeton, 1835)	Introduced (New to FLO)	602
Entognatha	Collembola	Entomobryidae	*Entomobrya atrocincta* Schött, 1897	Introduced	113
Entognatha	Collembola	Entomobryidae	*Entomobrya multifasciata* (Tullberg, 1871)	Introduced *	7162
Entognatha	Collembola	Entomobryidae	*Entomobrya nivalis* (Linnaeus, 1758)	Introduced	1621
Entognatha	Collembola	Entomobryidae	*Entomobrya regularis* Stach, 1963	Introduced (New to Azores: SMG)	5
Entognatha	Collembola	Entomobryidae	*Entomobrya* spp. (Potentially several species)	*	1885
Entognatha	Collembola	Entomobryidae	Entomobryidae sp. Nr. 1		105
Entognatha	Collembola	Entomobryidae	Entomobryidae sp. Nr. 2		397
Entognatha	Collembola	Entomobryidae	Entomobryidae sp. Nr. 3		91
Entognatha	Collembola	Entomobryidae	Entomobryidae sp. Nr. 4		1
Entognatha	Collembola	Entomobryidae	Entomobryidae sp. Nr. 5		19
Entognatha	Collembola	Entomobryidae	Entomobryidae sp. Nr. 6		96
Entognatha	Collembola	Entomobryidae	Entomobryidae sp. Nr. 7		41
Entognatha	Collembola	Entomobryidae	Entomobryidae sp. Nr. 8		70
Entognatha	Collembola	Entomobryidae	Entomobryidae sp. Nr. 9		4
Entognatha	Collembola	Entomobryidae	Entomobryidae sp. Nr. 10		31
Entognatha	Collembola	Entomobryidae	Entomobryidae sp. Nr. 11		60
Entognatha	Collembola	Entomobryidae	Entomobryidae sp. Nr. 12		222
Entognatha	Collembola	Entomobryidae	Entomobryidae sp. Nr. 13		723
Entognatha	Collembola	Entomobryidae	Entomobryidae sp. Nr. 14		97
Entognatha	Collembola	Entomobryidae	Entomobryidae sp. Nr. 15		12
Entognatha	Collembola	Entomobryidae	Entomobryidae sp. Nr. 16		955
Entognatha	Collembola	Entomobryidae	*Heteromurus* sp.	*	8430
Entognatha	Collembola	Entomobryidae	*Himalanura* sp.	Introduced (New genus to Azores)	45
Entognatha	Collembola	Entomobryidae	*Lepidocyrtus curvicollis* Bourlet, 1839	Introduced *	2844
Entognatha	Collembola	Entomobryidae	*Lepidocyrtus cyaneus* Tullberg, 1871	Introduced (New to FLO, PIC, TER)	773
Entognatha	Collembola	Entomobryidae	*Lepidocyrtus lusitanicus piezoensis* (Simón-Benito, 2007)	Introduced (New to Azores: TER, SMG, SMR)	237
Entognatha	Collembola	Entomobryidae	*Lepidocyrtus* sp.		39
Entognatha	Collembola	Entomobryidae	*Pogonognathellus longicornis* (Müller, 1776)	Introduced (New to FLO, TER, SMR) *	2007
Entognatha	Collembola	Hypogastruridae	*Ceratophysella denticulata* (Bagnall, 1941)	Introduced (New to SMR) *	15403
Entognatha	Collembola	Isotomidae	*Desoria* sp.	Introduced	230
Entognatha	Collembola	Isotomidae	*Desoria trispinata* (MacGillivray, 1896)	Introduced (New to SMR) *	4085
Entognatha	Collembola	Isotomidae	*Folsomia* sp.		780
Entognatha	Collembola	Isotomidae	*Folsomides* sp.		1170
Entognatha	Collembola	Isotomidae	*Isotoma* sp.		730
Entognatha	Collembola	Isotomidae	Isotomidae sp. Nr. 1		291
Entognatha	Collembola	Isotomidae	*Isotomurus palustris* (Müller, 1776)	Introduced (New to SMR)	113
Entognatha	Collembola	Isotomidae	*Isotomurus* spp. (potentially several species)		1644
Entognatha	Collembola	Isotomidae	*Folsomia* sp.		9
Entognatha	Collembola	Katiannidae	*Sminthurinus aureus* (Lubbock, 1862)	Introduced	16
Entognatha	Collembola	Katiannidae	*Sminthurinus elegans* (Fitch, 1863)	Introduced (New to FLO, PIC, SMR)	256
Entognatha	Collembola	Katiannidae	*Sminthurinus quadrimaculatus* (Ryder, 1879)	Introduced (New to Azores: TER, SMR)	6
Entognatha	Collembola	Neanuridae	Neanuridae sp. Nr. 1		20
Entognatha	Collembola	Neanuridae	Neanuridae sp. Nr. 2		17
Entognatha	Collembola	Onychiuridae	*Onychiurus* spp. (potentially more than one species)		1311
Entognatha	Collembola	Onychiuridae	*Protophorura* sp.	Introduced (New genus to Azores)	57
Entognatha	Collembola	Poduridae	*Neanura* sp.	Introduced	54
Entognatha	Collembola	Sminthuridae	*Bourletiella* sp.	Introduced	594
Entognatha	Collembola	Sminthuridae	*Dicyrtomina minuta* (O. Fabricius, 1783)	Introduced	208
Entognatha	Collembola	Sminthuridae	*Dicyrtomina ornata* (Nicolet, 1842)	Introduced (New to FLO, PIC, TER. SMR)	286
Entognatha	Collembola	Sminthuridae	*Dicyrtomina* sp.	Introduced	776
Entognatha	Collembola	Sminthuridae	*Jordanathrix articulata* (Ellis, 1974)	Introduced (New to Azores: FLO, PIC, TER, SMG, SMR)	1170
Entognatha	Collembola	Sminthuridae	*Jordanathrix* sp.		321
Entognatha	Collembola	Sminthuridae	*Lipothrix* sp.		136
Entognatha	Collembola	Sminthuridae	Sminthuridae sp. Nr. 1		46
Entognatha	Collembola	Sminthuridae	Sminthuridae sp. Nr. 2		249
Entognatha	Collembola	Sminthuridae	Sminthuridae sp. Nr. 3		374
Entognatha	Collembola	Sminthuridae	Sminthuridae sp. Nr. 4		49
Entognatha	Collembola	Sminthuridae	Sminthuridae sp. Nr. 5		4
Entognatha	Collembola	Sminthuridae	Sminthuridae sp. Nr. 6		304
Entognatha	Collembola	Sminthuridae	Sminthuridae sp. Nr. 7		994
Entognatha	Collembola	Sminthuridae	Sminthuridae sp. Nr. 8		32
Entognatha	Collembola	Sminthuridae	Sminthuridae sp. Nr. 9		15
Entognatha	Collembola	Sminthuridae	Sminthuridae sp. Nr. 10		40
Entognatha	Collembola	Sminthuridae	Sminthuridae sp. Nr. 11		16
Entognatha	Collembola	Sminthuridae	Sminthuridae sp. Nr. 12		508
Entognatha	Collembola	Sminthuridae	Sminthuridae sp. Nr. 13		134
Entognatha	Collembola	Sminthuridae	Sminthuridae sp. Nr. 14		1
Entognatha	Collembola	Sminthuridae	Sminthuridae sp. Nr. 15		9
Entognatha	Collembola	Sminthuridae	Sminthuridae sp. Nr. 16		374
Entognatha	Collembola	Sminthuridae	Sminthuridae sp. Nr. 17		23
Entognatha	Collembola	Sminthuridae	Sminthuridae sp. Nr. 18		12
Entognatha	Collembola	Sminthuridae	Sminthuridae sp. Nr. 19		109
Entognatha	Collembola	Sminthuridae	Sminthuridae sp. Nr. 20		189
Entognatha	Collembola	Sminthuridae	Sminthuridae sp. Nr. 21		31
Entognatha	Collembola	Sminthuridae	Sminthuridae sp. Nr. 22		11
Entognatha	Collembola	Sminthuridae	Sminthuridae sp. Nr. 23		4
Entognatha	Collembola	Sminthuridae	Sminthuridae sp. Nr. 24		2
Entognatha	Collembola	Sminthuridae	Sminthuridae sp. Nr. 25		7
Entognatha	Collembola	Sminthuridae	Sminthuridae sp. Nr. 26		1
Entognatha	Collembola	Sminthuridae	Sminthuridae sp. Nr. 27		521
Entognatha	Collembola	Sminthuridae	Sminthuridae sp. Nr. 28		63
Entognatha	Collembola	Sminthuridae	Sminthuridae sp. Nr. 29		72
Entognatha	Collembola	Sminthuridae	Sminthuridae sp. Nr. 30		130
Entognatha	Collembola	Sminthuridae	Sminthuridae sp. Nr. 31		13
Entognatha	Collembola	Sminthuridae	Sminthuridae sp. Nr. 32		9
Entognatha	Collembola	Sminthuridae	Sminthuridae sp. Nr. 33		1
Entognatha	Collembola	Sminthuridae	Sminthuridae sp. Nr. 34		3
Entognatha	Collembola	Sminthuridae	*Sminthurus viridis* (Linnaeus, 1758)	Introduced (New to FLO, PIC, TER)	775
Insecta	Coleoptera	Anobiidae	*Anobium punctatum* (De Geer, 1774)	Introduced	52
Insecta	Coleoptera	Anthicidae	*Hirticollis quadriguttatus* (Rossi, 1794)	Native (New to PIC)	94
Insecta	Coleoptera	Anthicidae	*Hirticomus* sp.		35
Insecta	Coleoptera	Carabidae	*Agonum muelleri muelleri* (Herbst, 1784)	Introduced	2
Insecta	Coleoptera	Carabidae	*Amara aenea* (De Geer, 1774)	Introduced	10
Insecta	Coleoptera	Carabidae	*Anisodactylus binotatus* (Fabricius, 1787)	Introduced	3
Insecta	Coleoptera	Carabidae	*Asaphidion flavipes* (Linnaeus, 1761)	Introduced (New to Azores: SMG)	1
Insecta	Coleoptera	Carabidae	*Harpalus distinguendus distinguendus* (Duftschmidt, 1812)	Introduced	1
Insecta	Coleoptera	Carabidae	*Harpalus* sp.	Introduced	5
Insecta	Coleoptera	Carabidae	*Microlestes negrita negrita* (Wollaston, 1854)	Native (New to FLO, PIC)	15
Insecta	Coleoptera	Carabidae	*Notiophilus quadripunctatus* Dejean, 1826	Native (New to FLO, PIC)	3
Insecta	Coleoptera	Carabidae	*Ocys harpaloides* (Audinet-Serville, 1821)	Native	58
Insecta	Coleoptera	Carabidae	*Pseudoophonus rufipes* (De Geer, 1774)	Introduced	138
Insecta	Coleoptera	Carabidae	*Pterostichus vernalis* (Panzer, 1796)	Introduced	26
Insecta	Coleoptera	Carabidae	*Stenolophus teutonus* (Schrank, 1781)	Native	1
Insecta	Coleoptera	Chrysomelidae	*Chaetocnema hortensis* (Fourcroy, 1785)	Introduced	41
Insecta	Coleoptera	Chrysomelidae	*Chrysolina bankii* (Fabricius, 1775)	Native	81
Insecta	Coleoptera	Chrysomelidae	*Epitrix* sp.		43
Insecta	Coleoptera	Chrysomelidae	*Longitarsus kutscherae* (Rye, 1872)	Introduced (New to PIC)	38
Insecta	Coleoptera	Chrysomelidae	*Longitarsus lateripunctatus lateripunctatus* (Rosenhauer, 1856)	Introduced	6
Insecta	Coleoptera	Coccinellidae	*Adalia decempunctata* (Linnaeus, 1758)	Introduced	1
Insecta	Coleoptera	Coccinellidae	*Clitostethus arcuatus* (Rossi, 1794)	Introduced (New to PIC)	41
Insecta	Coleoptera	Coccinellidae	*Coccinella undecimpunctata undecimpunctata* Linnaeus, 1758	Introduced	4
Insecta	Coleoptera	Coccinellidae	*Nephus* sp.		4
Insecta	Coleoptera	Coccinellidae	*Nephus voeltzkowi* (Weise, 1910)	Introduced	4
Insecta	Coleoptera	Coccinellidae	*Rodolia cardinalis* (Mulsant, 1850)	Introduced	14
Insecta	Coleoptera	Coccinellidae	*Scymnus interruptus* (Goeze, 1777)	Native	24
Insecta	Coleoptera	Coccinellidae	*Scymnus* sp.		427
Insecta	Coleoptera	Coccinellidae	*Stethorus pusillus* (Herbst, 1979)	Native (New to FLO, PIC)	71
Insecta	Coleoptera	Corylophidae	*Sericoderus lateralis* (Gyllenhal, 1827)	Introduced	903
Insecta	Coleoptera	Curculionidae	*Calacalles subcarinatus* (Israelson, 1984)	Endemic	21
Insecta	Coleoptera	Curculionidae	*Coccotrypes carpophagus* (Hornung, 1842)	Introduced	66
Insecta	Coleoptera	Curculionidae	*Drouetius* sp.	Endemic	51
Insecta	Coleoptera	Curculionidae	*Mecinus pascuorum* Gyllenhal, 1813	Introduced (New to FLO, SMG)	30
Insecta	Coleoptera	Curculionidae	*Orthochaetes insignis* (Aubé, 1863)	Native	7
Insecta	Coleoptera	Curculionidae	*Otiorhynchus cribricollis* Gyllenhal, 1834	Introduced	109
Insecta	Coleoptera	Curculionidae	*Pseudechinosoma nodosum* Hustache, 1936	Endemic	5
Insecta	Coleoptera	Curculionidae	*Pseudocaulotrupis parvus* (Israelson, 1985)	Endemic	25
Insecta	Coleoptera	Curculionidae	*Pseudocaulotrupis* sp.	Endemic	15
Insecta	Coleoptera	Curculionidae	*Xyleborus alni* Nijima, 1909	Introduced	14
Insecta	Coleoptera	Dryophthoridae	*Sitophilus* sp.	Introduced	39
Insecta	Coleoptera	Elateridae	*Heteroderes azoricus* (Tarnier, 1860)	Endemic	234
Insecta	Coleoptera	Hydrophilidae	*Cercyon haemorrhoidalis* (Fabricius, 1775)	Introduced	7
Insecta	Coleoptera	Latridiidae	*Metophthalmus occidentalis* Israelson, 1984	Endemic	4
Insecta	Coleoptera	Mycetophagidae	*Typhaea stercorea* (Linnaeus, 1758)	Introduced	97
Insecta	Coleoptera	Nitidulidae	*Brassicogethes aeneus* (Fabricius, 1775)	Introduced	5
Insecta	Coleoptera	Nitidulidae	*Carpophilus fumatus* (Boheman, 1851)	Introduced	10
Insecta	Coleoptera	Nitidulidae	*Carpophilus hemipterus* (Linnaeus, 1758)	Introduced	3
Insecta	Coleoptera	Nitidulidae	*Carpophilus* spp. (possibly more than one species)	Introduced	20
Insecta	Coleoptera	Nitidulidae	*Epuraea biguttata* (Thunberg, 1784)	Introduced	703
Insecta	Coleoptera	Nitidulidae	*Epuraea* sp.	Introduced	51
Insecta	Coleoptera	Nitidulidae	*Stelidota geminata* (Say, 1825)	Introduced	35
Insecta	Coleoptera	Phalacridae	*Stilbus testaceus* (Panzer, 1797)	Native	84
Insecta	Coleoptera	Ptiliidae	*Ptenidium pusillum* (Gyllenhal, 1808)	Introduced	36
Insecta	Coleoptera	Ptinidae	*Sphaericus* sp.	(blank)	1
Insecta	Coleoptera	Scarabaeidae	*Calamosternus granarius* (Linnaeus, 1767)	Introduced	19
Insecta	Coleoptera	Scarabaeidae	*Onthophagus taurus* (Schreber,1759)	Introduced	4
Insecta	Coleoptera	Scarabaeidae	*Popillia japonica* Newman, 1838	Introduced (New to PIC)	168
Insecta	Coleoptera	Scraptiidae	*Anaspis proteus* Wollaston, 1854	Native	46
Insecta	Coleoptera	Scydmaenidae	*Euconnus* sp.		18
Insecta	Coleoptera	Silvanidae	*Cryptamorpha desjardinsii* (Guérin-Méneville, 1844)	Introduced	94
Insecta	Coleoptera	Silvanidae	*Silvanus* sp.		36
Insecta	Coleoptera	Staphylinidae	*Aleochara clavicornis* Redtenbacher, 1849	Introduced	4
Insecta	Coleoptera	Staphylinidae	*Aleochara* sp.		18
Insecta	Coleoptera	Staphylinidae	*Anotylus nitidifrons* (Wollaston, 1871)	Introduced	360
Insecta	Coleoptera	Staphylinidae	*Astenus lyonessius* (Joy, 1908)	Native	7
Insecta	Coleoptera	Staphylinidae	*Atheta atramentaria* (Gyllenhal, 1810)	Introduced	39
Insecta	Coleoptera	Staphylinidae	*Atheta fungi* (Gravenhorst, 1806)	Introduced	573
Insecta	Coleoptera	Staphylinidae	*Atheta* sp. (possibly more than one species)		77
Insecta	Coleoptera	Staphylinidae	*Carpelimus corticinus* (Gravenhorst, 1806)	Native	2
Insecta	Coleoptera	Staphylinidae	*Coproporus pulchellus* (Erichson, 1839)	Introduced	7
Insecta	Coleoptera	Staphylinidae	*Cordalia obscura* (Gravenhorst, 1802)	Introduced	58
Insecta	Coleoptera	Staphylinidae	*Euplectus infirmus* (Raffray, 1910)	Introduced (New to PIC, SMR)	5
Insecta	Coleoptera	Staphylinidae	*Gabrius nigritulus* (Gravenhorst, 1802)	Introduced	58
Insecta	Coleoptera	Staphylinidae	*Gyrohypnus fracticornis* (Müller, 1776)	Introduced	7
Insecta	Coleoptera	Staphylinidae	*Medon* sp.		4
Insecta	Coleoptera	Staphylinidae	*Ocypus aethiops* (Waltl, 1835)	Native	15
Insecta	Coleoptera	Staphylinidae	*Ocypus olens* (Müller, 1764)	Native	11
Insecta	Coleoptera	Staphylinidae	*Oligota pumilio* Kiesenwetter, 1858	Native (New to FLO, PIC)	42
Insecta	Coleoptera	Staphylinidae	*Oligota* sp.		3
Insecta	Coleoptera	Staphylinidae	*Oxytelus sculptus* Gravenhorst, 1806	Introduced (New to PIC)	29
Insecta	Coleoptera	Staphylinidae	*Philonthus* sp.		8
Insecta	Coleoptera	Staphylinidae	*Phloeonomus punctipennis* Thomson, 1867	Native	1
Insecta	Coleoptera	Staphylinidae	*Phloeonomus* sp.	(blank)	75
Insecta	Coleoptera	Staphylinidae	*Proteinus atomarius* Erichson, 1840	Native	45
Insecta	Coleoptera	Staphylinidae	*Quedius curtipennis* Bernhauer, 1908	Native (New to PIC)	22
Insecta	Coleoptera	Staphylinidae	*Quedius simplicifrons* Fairmaire, 1862	Native (New to FLO, SMG)	42
Insecta	Coleoptera	Staphylinidae	*Rugilus orbiculatus* (Paykull, 1789)	Native	113
Insecta	Coleoptera	Staphylinidae	*Sepedophilus lusitanicus* Hammond, 1973	Native	9
Insecta	Coleoptera	Staphylinidae	*Tachyporus chrysomelinus* (Linnaeus, 1758)	Introduced	8
Insecta	Coleoptera	Staphylinidae	*Tachyporus dispar* (Paykull, 1789)	Introduced (New to Azores: FLO, SMG)	5
Insecta	Coleoptera	Staphylinidae	*Tachyporus nitidulus* (Fabricius, 1781)	Introduced	6
Insecta	Coleoptera	Staphylinidae	*Xantholinus longiventris* Heer, 1839	Introduced (New to FLO)	7
Insecta	Coleoptera	Zopheridae	*Tarphius floresensis* Borges & Serrano, 2017	Endemic	1
Insecta	Coleoptera	Zopheridae	*Tarphius rufonodulosus* Israelson, 1984	Endemic	1
Insecta	Dermaptera	Anisolabidae	*Euborellia annulipes* (Lucas, 1847)	Introduced	540
Insecta	Dermaptera	Forficulidae	*Forficula auricularia* Linnaeus, 1758	Introduced	32
Insecta	Hemiptera	Aphididae	*Aphis fabae* Scopoli, 1763	Introduced (New to PIC)	333
Insecta	Hemiptera	Aphididae	*Aphis gossypii* Glover, 1877	Native	19
Insecta	Hemiptera	Aphididae	*Aulacorthum solani* (Kaltenbach, 1843)	Native (New to PIC)	1566
Insecta	Hemiptera	Aphididae	*Cinara juniperi* (De Geer, 1773)	Native *	4261
Insecta	Hemiptera	Cicadellidae	*Anoscopus albifrons* (Linnaeus, 1758)	Native	59
Insecta	Hemiptera	Cicadellidae	*Aphrodes hamiltoni* Quartau & Borges, 2003	Endemic	100
Insecta	Hemiptera	Cixiidae	*Cixius* spp. (several potential species and subspecies)	Endemic	674
Insecta	Hemiptera	Coccidae	*Protopulvinaria pyriformis* (Cockerell, 1894)	Introduced	361
Insecta	Hemiptera	Cydnidae	*Geotomus punctulatus* (A. Costa, 1847)	Native	32
Insecta	Hemiptera	Delphacidae	*Megamelodes quadrimaculatus* (Signoret, 1865)	Native	220
Insecta	Hemiptera	Diaspididae	*Aspidiotus nerii* Bouché, 1833	Introduced	2
Insecta	Hemiptera	Diaspididae	*Chrysomphalus pinnulifer* (Maskell, 1891)	Native (New to PIC, SMR)	116
Insecta	Hemiptera	Flatidae	*Cyphopterum adcendens* (Herrich-Schaeffer, 1835)	Native	1219
Insecta	Hemiptera	Lygaeidae	*Kleidocerys ericae* (Horváth, 1908)	Native	18
Insecta	Hemiptera	Margarodidae	*Icerya purchasi* Maskell, 1878	Introduced (New to FLO, SMR)	609
Insecta	Hemiptera	Miridae	*Monalocoris filicis* (Linnaeus, 1758)	Native	254
Insecta	Hemiptera	Miridae	*Pinalitus oromii* J. Ribes, 1992	Endemic	88
Insecta	Hemiptera	Pentatomidae	*Acrosternum heegeri* Fieber, 1861	Introduced (New to Azores: FLO, TER)	6
Insecta	Hemiptera	Reduviidae	*Empicoris rubromaculatus* (Blackburn, 1889)	Introduced (New to FLO)	27
Insecta	Hemiptera	Stenocephalidae	*Dicranocephalus agilis* (Scopoli, 1763)	Native (New to PIC)	2
Insecta	Hemiptera	Triozidae	*Trioza laurisilvae* Hodkinson, 1990	Native	70
Insecta	Hymenoptera	Aphelinidae	Aphelinidae sp. Nr. 1		72
Insecta	Hymenoptera	Aphelinidae	Aphelinidae sp. Nr. 2		4
Insecta	Hymenoptera	Aphelinidae	Aphelinidae sp. Nr. 3		3
Insecta	Hymenoptera	Aphelinidae	Aphelinidae sp. Nr. 4		44
Insecta	Hymenoptera	Aphelinidae	Aphelinidae sp. Nr. 5		43
Insecta	Hymenoptera	Aphelinidae	*Encarsia citrina* (Crawford, 1891)	Native (New to FLO, PIC)	5
Insecta	Hymenoptera	Aphelinidae	*Encarsia* sp.		2
Insecta	Hymenoptera	Apidae	*Apis mellifera* Linnaeus, 1758	Introduced	9
Insecta	Hymenoptera	Apidae	*Bombus ruderatus* (Fabricius, 1775)	Introduced	6
Insecta	Hymenoptera	Argidae	Argidae sp.		3
Insecta	Hymenoptera	Bethylidae	Gen. sp.		2
Insecta	Hymenoptera	Braconidae	*Aphaereta difficilis* Nixon, 1939	Native (New to FLO, PIC, TER, SMR)	615
Insecta	Hymenoptera	Braconidae	*Aphaereta* sp.1		36
Insecta	Hymenoptera	Braconidae	*Aphaereta* sp.2		30
Insecta	Hymenoptera	Braconidae	*Aphaereta* sp.3		46
Insecta	Hymenoptera	Braconidae	*Aphidius colemani* Viereck, 1912	Introduced (New to Azores: PIC, TER, SMG)	13
Insecta	Hymenoptera	Braconidae	*Aphidius ervi* Haliday, 1834	Introduced (New to Azores: PIC, TER)	33
Insecta	Hymenoptera	Braconidae	*Aphidius matricariae* Viereck, 1912	Introduced (New to Azores: PIC, TER)	6
Insecta	Hymenoptera	Braconidae	*Aphidius rhopalosiphi* Stefani-Perez, 1902	Introduced (New to Azores: PIC, TER, SMG, SMR)	18
Insecta	Hymenoptera	Braconidae	*Aphidius rosae* (Haliday, 1834)	Introduced (New to Azores: PIC)	1
Insecta	Hymenoptera	Braconidae	*Aphidius* sp.		21
Insecta	Hymenoptera	Braconidae	*Aphidius urticae* Haliday, 1834	Introduced (New to Azores: FLO, PIC, TER, SMG)	15
Insecta	Hymenoptera	Braconidae	*Aspilota* spp. (potentially four species)	Introduced (New genus to Azores)	49
Insecta	Hymenoptera	Braconidae	*Bassus rugulosus* (Nees, 1834)	Native (New to FLO, PIC)	10
Insecta	Hymenoptera	Braconidae	*Bracon intercessor* Nees, 1834	Native (New to PIC)	7
Insecta	Hymenoptera	Braconidae	Braconidae sp. Nr. 1		1
Insecta	Hymenoptera	Braconidae	Braconidae sp. Nr. 2		13
Insecta	Hymenoptera	Braconidae	*Centistidea ectoedemiae* Rohwer, 1914	Introduced (New to Azores: FLO, PIC, TER, SMR)	10
Insecta	Hymenoptera	Braconidae	*Chorebus* spp. (Potentially four species)	Introduced (New genus to Azores)	175
Insecta	Hymenoptera	Braconidae	*Dapsilarthra* sp.	Introduced	1
Insecta	Hymenoptera	Braconidae	*Dinotrema* sp.	Introduced	29
Insecta	Hymenoptera	Braconidae	Braconidae sp.		5
Insecta	Hymenoptera	Braconidae	*Homolobus* sp.	Introduced	9
Insecta	Hymenoptera	Braconidae	*Lodbrokia* sp.	Introduced (New genus to Azores)	13
Insecta	Hymenoptera	Braconidae	*Lysiphlebus fabarum* (Marshall, 1896)	Native (New to FLO, PIC)	16
Insecta	Hymenoptera	Braconidae	*Lysiphlebus testaceipes* (Cresson, 1880)	Native (New to FLO, PIC, SMG)	19
Insecta	Hymenoptera	Braconidae	*Macrocentrus collaris* (Spinola, 1808)	Native (New to PIC, TER)	5
Insecta	Hymenoptera	Braconidae	*Meteorus collaris* (Spin.) Hal. – Ruschka, Fulmek, 1915	Introduced (New to Azores: PIC, TER)	3
Insecta	Hymenoptera	Braconidae	*Meteorus unicolor* (Wesmael, 1835)	Introduced (New to Azores: FLO, PIC, TER)	18
Insecta	Hymenoptera	Braconidae	*Microgaster* sp.	New genus to Azores	5
Insecta	Hymenoptera	Braconidae	*Opius* sp.	Introduced	109
Insecta	Hymenoptera	Braconidae	*Orthostigmceratoa cratospilum* (Thomson, 1895)	Introduced (New to Azores: PIC, TER, SMG)	5
Insecta	Hymenoptera	Braconidae	*Orthostigma latriventris* Ratzeburg, 1844	Introduced (New to Azores: PIC, TER, SMR)	8
Insecta	Hymenoptera	Braconidae	*Orthostigma* spp.	Introduced (Potentially new records to Azores)	179
Insecta	Hymenoptera	Braconidae	*Pentapleura pumilio* (Nees, 1812)	Introduced (New to FLO, PIC; SMG)	295
Insecta	Hymenoptera	Braconidae	*Pentapleura* spp. (Potentially several species)	Introduced	39
Insecta	Hymenoptera	Braconidae	*Pseudopezomachus bituberculatus* (Marshall, 1905)	Introduced (New to Azores: PIC, SMG)	2
Insecta	Hymenoptera	Braconidae	*Tanycarpa punctata* (van Achterberg, 1976)	Introduced (New to Azores: SMG)	1
Insecta	Hymenoptera	Chalcididae	Gen. sp.		10
Insecta	Hymenoptera	Chalcidoidea	Chalcidoidea sp. Nr. 1		273
Insecta	Hymenoptera	Chalcidoidea	Chalcidoidea sp. Nr. 10		7
Insecta	Hymenoptera	Chalcidoidea	Chalcidoidea sp. Nr. 11		6
Insecta	Hymenoptera	Chalcidoidea	Chalcidoidea sp. Nr. 12		1
Insecta	Hymenoptera	Chalcidoidea	Chalcidoidea sp. Nr. 2		112
Insecta	Hymenoptera	Chalcidoidea	Chalcidoidea sp. Nr. 3		52
Insecta	Hymenoptera	Chalcidoidea	Chalcidoidea sp. Nr. 4		38
Insecta	Hymenoptera	Chalcidoidea	Chalcidoidea sp. Nr. 5		4
Insecta	Hymenoptera	Chalcidoidea	Chalcidoidea sp. Nr. 6		2
Insecta	Hymenoptera	Chalcidoidea	Chalcidoidea sp. Nr. 7		167
Insecta	Hymenoptera	Chalcidoidea	Chalcidoidea sp. Nr. 8		26
Insecta	Hymenoptera	Chalcidoidea	Chalcidoidea sp. Nr. 9		273
Insecta	Hymenoptera	Chrysididae	*Chrysis ignita ignita* (Linnaeus, 1758)	Native (New to FLO)	1
Insecta	Hymenoptera	Cynipidae	Cynipidae sp. Nr. 2		25
Insecta	Hymenoptera	Cynipidae	Cynipidae sp. Nr. 3		86
Insecta	Hymenoptera	Cynipidae	Cynipidae ssp. Nr. 1		26
Insecta	Hymenoptera	Diapriidae	Diapriidae sp. Nr.		4
Insecta	Hymenoptera	Diapriidae	Diapriidae sp. Nr. 1		242
Insecta	Hymenoptera	Diapriidae	Diapriidae sp. Nr. 10		25
Insecta	Hymenoptera	Diapriidae	Diapriidae sp. Nr. 2		57
Insecta	Hymenoptera	Diapriidae	Diapriidae sp. Nr. 3		77
Insecta	Hymenoptera	Diapriidae	Diapriidae sp. Nr. 4		144
Insecta	Hymenoptera	Diapriidae	Diapriidae sp. Nr. 5		43
Insecta	Hymenoptera	Diapriidae	Diapriidae sp. Nr. 6		34
Insecta	Hymenoptera	Diapriidae	Diapriidae sp. Nr. 7		78
Insecta	Hymenoptera	Diapriidae	Diapriidae sp. Nr. 8		97
Insecta	Hymenoptera	Diapriidae	Diapriidae sp. Nr. 9		8
Insecta	Hymenoptera	Dryinidae	*Gonatopus clavipes* (Thunberg, 1827)	Introduced (New to Azores: SMG)	1
Insecta	Hymenoptera	Elasmidae	Gen. sp.		26
Insecta	Hymenoptera	Encyrtidae	Encyrtidae sp. Nr. 1		13
Insecta	Hymenoptera	Encyrtidae	Encyrtidae sp. Nr. 2		1411
Insecta	Hymenoptera	Encyrtidae	Encyrtidae sp. Nr. 3		11
Insecta	Hymenoptera	Encyrtidae	Encyrtidae sp. Nr. 4		3
Insecta	Hymenoptera	Encyrtidae	Encyrtidae sp. Nr. 5		5
Insecta	Hymenoptera	Encyrtidae	Encyrtidae sp. Nr. 6		78
Insecta	Hymenoptera	Encyrtidae	Encyrtidae sp. Nr. 7		18
Insecta	Hymenoptera	Encyrtidae	Encyrtidae sp. Nr. 8		27
Insecta	Hymenoptera	Encyrtidae	Encyrtidae sp. Nr. 9		159
Insecta	Hymenoptera	Encyrtidae	Encyrtidae sp. Nr. 10		11
Insecta	Hymenoptera	Encyrtidae	Encyrtidae sp. Nr. 11		161
Insecta	Hymenoptera	Encyrtidae	Encyrtidae sp. Nr. 12		55
Insecta	Hymenoptera	Encyrtidae	Encyrtidae sp. Nr. 13		41
Insecta	Hymenoptera	Encyrtidae	Encyrtidae sp. Nr. 14		78
Insecta	Hymenoptera	Encyrtidae	Encyrtidae sp. Nr. 15		209
Insecta	Hymenoptera	Encyrtidae	Encyrtidae sp. Nr. 16		28
Insecta	Hymenoptera	Encyrtidae	Encyrtidae sp. Nr. 17		20
Insecta	Hymenoptera	Encyrtidae	Encyrtidae sp. Nr. 18		17
Insecta	Hymenoptera	Encyrtidae	Encyrtidae sp. Nr. 19		9
Insecta	Hymenoptera	Encyrtidae	Encyrtidae sp. Nr. 20		3
Insecta	Hymenoptera	Encyrtidae	Encyrtidae sp. Nr. 21		18
Insecta	Hymenoptera	Encyrtidae	Encyrtidae sp. Nr. 22		1
Insecta	Hymenoptera	Encyrtidae	Encyrtidae sp. Nr. 23		62
Insecta	Hymenoptera	Encyrtidae	Encyrtidae sp. Nr. 24		58
Insecta	Hymenoptera	Encyrtidae	Encyrtidae sp. Nr. 25		3
Insecta	Hymenoptera	Encyrtidae	Encyrtidae sp. Nr. 26		298
Insecta	Hymenoptera	Encyrtidae	Encyrtidae sp. Nr. 27		16
Insecta	Hymenoptera	Encyrtidae	Encyrtidae sp. Nr. 28		5
Insecta	Hymenoptera	Encyrtidae	Encyrtidae sp. Nr. 29		51
Insecta	Hymenoptera	Encyrtidae	Encyrtidae sp. Nr. 30		9
Insecta	Hymenoptera	Encyrtidae	Encyrtidae sp. Nr. 31		58
Insecta	Hymenoptera	Encyrtidae	Encyrtidae sp. Nr. 32		8
Insecta	Hymenoptera	Encyrtidae	Encyrtidae sp. Nr. 33		8
Insecta	Hymenoptera	Encyrtidae	Encyrtidae sp. Nr. 34		106
Insecta	Hymenoptera	Encyrtidae	Encyrtidae sp. Nr. 35		1
Insecta	Hymenoptera	Encyrtidae	Encyrtidae sp. Nr. 36		2
Insecta	Hymenoptera	Encyrtidae	Encyrtidae sp. Nr. 37		9
Insecta	Hymenoptera	Encyrtidae	Encyrtidae sp. Nr. 38		18
Insecta	Hymenoptera	Encyrtidae	Encyrtidae sp. Nr. 39		46
Insecta	Hymenoptera	Encyrtidae	Encyrtidae sp. Nr. 40		31
Insecta	Hymenoptera	Encyrtidae	Encyrtidae sp. Nr. 41		60
Insecta	Hymenoptera	Encyrtidae	Encyrtidae sp. Nr. 42		387
Insecta	Hymenoptera	Encyrtidae	Encyrtidae sp. Nr. 43		2
Insecta	Hymenoptera	Encyrtidae	Encyrtidae sp. Nr. 44		5
Insecta	Hymenoptera	Encyrtidae	Encyrtidae sp. Nr. 45		89
Insecta	Hymenoptera	Encyrtidae	Encyrtidae sp. Nr. 46		13
Insecta	Hymenoptera	Encyrtidae	Encyrtidae sp. Nr. 47		16
Insecta	Hymenoptera	Encyrtidae	Encyrtidae sp. Nr. 48		2
Insecta	Hymenoptera	Encyrtidae	Encyrtidae sp. Nr. 49		26
Insecta	Hymenoptera	Encyrtidae	Encyrtidae sp. Nr. 50		218
Insecta	Hymenoptera	Encyrtidae	Eulophidae sp. Nr. 1		25
Insecta	Hymenoptera	Encyrtidae	Eulophidae sp. Nr. 2		7
Insecta	Hymenoptera	Encyrtidae	Eulophidae sp. Nr. 3		3
Insecta	Hymenoptera	Encyrtidae	Eulophidae sp. Nr. 4		11
Insecta	Hymenoptera	Eupelmidae	Gen. sp.		131
Insecta	Hymenoptera	Figitidae	Gen. sp.		70
Insecta	Hymenoptera	Formicidae	*Lasius grandis* Forel, 1909	Native *	15469
Insecta	Hymenoptera	Formicidae	*Temnothorax unifasciatus* (Latreille, 1798)	Native	5
Insecta	Hymenoptera	Ichneumonidae	Gen. sp.		18
Insecta	Hymenoptera	Ichneumonidae	Ichneumonidae sp. Nr. 1		94
Insecta	Hymenoptera	Ichneumonidae	Ichneumonidae sp. Nr. 2		5
Insecta	Hymenoptera	Ichneumonidae	Ichneumonidae sp. Nr. 3		10
Insecta	Hymenoptera	Ichneumonidae	Ichneumonidae sp. Nr. 4		26
Insecta	Hymenoptera	Ichneumonidae	Ichneumonidae sp. Nr. 5		24
Insecta	Hymenoptera	Ichneumonidae	Ichneumonidae sp. Nr. 6		3
Insecta	Hymenoptera	Ichneumonidae	Ichneumonidae sp. Nr. 7		6
Insecta	Hymenoptera	Ichneumonidae	Ichneumonidae sp. Nr. 8		14
Insecta	Hymenoptera	Ichneumonoidea	Ichneumonoidea sp. Nr. 1		8
Insecta	Hymenoptera	Ichneumonoidea	Ichneumonoidea sp. Nr. 2		6
Insecta	Hymenoptera	Ichneumonoidea	Ichneumonoidea sp. Nr. 3		33
Insecta	Hymenoptera	Ichneumonoidea	Ichneumonoidea sp. Nr. 4		35
Insecta	Hymenoptera	Ichneumonoidea	Ichneumonoidea sp. Nr. 5		29
Insecta	Hymenoptera	Ichneumonoidea	Ichneumonoidea sp. Nr. 6		2
Insecta	Hymenoptera	Ichneumonoidea	Ichneumonoidea sp. Nr. 7		2
Insecta	Hymenoptera	Ichneumonoidea	Ichneumonoidea sp. Nr. 8		5
Insecta	Hymenoptera	Megachilidae	Gen. sp.		14
Insecta	Hymenoptera	Megaspilidae	Gen. sp.		26
Insecta	Hymenoptera	Mymaridae	*Mymar taprobanicum* Ward, 1875	Native (New to FLO, PIC, TER, SMG, SMR)	289
Insecta	Hymenoptera	Mymaridae	Mymaridae sp. Nr. 1		1
Insecta	Hymenoptera	Mymaridae	Mymaridae sp. Nr. 2		23
Insecta	Hymenoptera	Mymaridae	Mymaridae sp. Nr. 3		4
Insecta	Hymenoptera	Mymaridae	Mymaridae sp. Nr. 4		14
Insecta	Hymenoptera	Mymaridae	Mymaridae sp. Nr. 5		6
Insecta	Hymenoptera	Mymaridae	Mymaridae sp. Nr. 6		2
Insecta	Hymenoptera	Proctotrupidae	Proctotrupidae sp. Nr. 1		48
Insecta	Hymenoptera	Proctotrupidae	Proctotrupidae sp. Nr. 2		13
Insecta	Hymenoptera	Proctotrupidae	Proctotrupidae sp. Nr. 3		2
Insecta	Hymenoptera	Proctotrupidae	Proctotrupidae sp. Nr. 4		1
Insecta	Hymenoptera	Proctotrupidae	Proctotrupidae sp. Nr. 5		7
Insecta	Hymenoptera	Pteromalidae	*Pycnetron* sp.		25
Insecta	Hymenoptera	Scelionidae	Scelionidae sp. Nr. 1		19
Insecta	Hymenoptera	Scelionidae	Scelionidae sp. Nr. 2		29
Insecta	Hymenoptera	Scelionidae	Scelionidae sp. Nr. 3		20
Insecta	Hymenoptera	Scelionidae	Scelionidae sp. Nr. 4		3
Insecta	Hymenoptera	Scelionidae	Scelionidae sp. Nr. 5		23
Insecta	Hymenoptera	Scelionidae	Scelionidae sp. Nr. 6		10
Insecta	Hymenoptera	Scelionidae	Scelionidae sp. Nr. 7		136
Insecta	Hymenoptera	Scelionidae	Scelionidae sp. Nr. 8		41
Insecta	Hymenoptera	Scelionidae	Scelionidae sp. Nr. 9		5
Insecta	Hymenoptera	Scelionidae	Scelionidae sp. Nr. 10		15
Insecta	Hymenoptera	Scelionidae	Scelionidae sp. Nr. 11		27
Insecta	Hymenoptera	Scelionidae	Scelionidae sp. Nr. 12		18
Insecta	Hymenoptera	Scelionidae	Scelionidae sp. Nr. 13		6
Insecta	Hymenoptera	Scelionidae	Scelionidae sp. Nr. 14		31
Insecta	Hymenoptera	Scelionidae	Scelionidae sp. Nr. 15		160
Insecta	Hymenoptera	Scelionidae	Scelionidae sp. Nr. 16		2
Insecta	Hymenoptera	Scelionidae	Scelionidae sp. Nr. 17		6
Insecta	Hymenoptera	Scelionidae	Scelionidae sp. Nr. 18		30
Insecta	Hymenoptera	Scelionidae	Scelionidae sp. Nr. 19		83
Insecta	Hymenoptera	Scelionidae	Scelionidae sp. Nr. 20		72
Insecta	Hymenoptera	Sphecidae	Gen. sp.		5
Insecta	Hymenoptera	Tetracampidae	Gen. sp.		178
Insecta	Neuroptera	Chrysopidae	*Chrysoperla lucasina* (Lacroix, 1912)	Introduced	9
Insecta	Neuroptera	Hemerobiidae	*Hemerobius azoricus* Tjeder, 1948	Endemic	3
Insecta	Neuroptera	Hemerobiidae	*Hemerobius humulinus* Linnaeus, 1758	Native	55
Insecta	Orthoptera	Gryllidae	*Gryllus bimaculatus* De Geer, 1773	Introduced	5
Insecta	Orthoptera	Gryllidae	*Gryllus* sp.		59
Insecta	Orthoptera	Tettigoniidae	*Neoconocephalus* sp.		16
Insecta	Psocoptera	Caeciliusidae	*Valenzuela flavidus* (Stephens, 1836)	Native	86
Insecta	Psocoptera	Ectopsocidae	*Ectopsocus briggsi* McLachlan, 1899	Introduced	694
Insecta	Psocoptera	Elipsocidae	*Elipsocus brincki* Badonnel, 1963	Endemic	6
Insecta	Psocoptera	Trichopsocidae	*Trichopsocus clarus* (Banks, 1908)	Native	39
Insecta	Thysanoptera	Phlaeothripidae	*Hoplothrips* sp.		446
Insecta	Thysanoptera	Thripidae	*Heliothrips haemorrhoidalis* (Bouché, 1833)	Introduced	564
